# Dynamic usage of transcription start sites within core promoters

**DOI:** 10.1186/gb-2006-7-12-r118

**Published:** 2006-12-12

**Authors:** Hideya Kawaji, Martin C Frith, Shintaro Katayama, Albin Sandelin, Chikatoshi Kai, Jun Kawai, Piero Carninci, Yoshihide Hayashizaki

**Affiliations:** 1 NTT Software Corporation, 209 Yamashita-cho Nakak-ku, Yokohama, Kanagawa, 231-8551, Japan; 2 Genome Exploration Research Group (Genome Network Project Core Group), RIKEN Genomic Sciences Center (GSC), RIKEN Yokohama Institute, 1-7-22 Suehiro-cho, Tsurumi-ku, Yokohama, Kanagawa, 230-0045, Japan; 3 Institute for Molecular Bioscience, University of Queensland, 306 Carmody Road, Brisbane, Queensland 4072, Australia; 4 The Bioinformatics Centre, University of Copenhagen, Universitetsparken 15, DK-2100 København Ø, Denmark; 5 Genome Science Laboratory, Discovery Research Institute, RIKEN Wako Institute, 2-1 Hirosawa, Wako, Saitama, 351-0198, Japan

## Abstract

**Background:**

Mammalian promoters do not initiate transcription at single, well defined base pairs, but rather at multiple, alternative start sites spread across a region. We previously characterized the static structures of transcription start site usage within promoters at the base pair level, based on large-scale sequencing of transcript 5' ends.

**Results:**

In the present study we begin to explore the internal dynamics of mammalian promoters, and demonstrate that start site selection within many mouse core promoters varies among tissues. We also show that this dynamic usage of start sites is associated with CpG islands, broad and multimodal promoter structures, and imprinting.

**Conclusion:**

Our results reveal a new level of biologic complexity within promoters - fine-scale regulation of transcription starting events at the base pair level. These events are likely to be related to epigenetic transcriptional regulation.

## Abstract 2

An exploration of the internal dynamics of mammalian promoters demonstrates that start site selection within mouse core promoters varies amongst tissues.

## Background

There is great interest in elucidating the control of transcription initiation, because these controls are major components of the gene regulatory networks that underlie the development and diversity of animals [1,2]. The standard view is that regulatory action takes place at distal and proximal enhancer and repressor *cis *elements, which are bound by transcription factors that interact with the basal transcription machinery at the core promoter to influence transcription. In this view, core promoters themselves are functionally simple, but recent data reveal that they are structurally complex, with a range of alternative transcription start sites (TSSs) at the base pair level [3-5]. A key issue is whether these complex structures are just 'biologic noise' from imprecise binding of basal transcription factors or whether TSS selection is precisely regulated.

Cap analysis of gene expression (CAGE) is a method used to identify TSSs and, at the same time, to measure their expression levels by counting a large number of sequenced 5' ends of full-length cDNAs, termed CAGE tags [6,7]. The advantage of this method is that it provides a view at base pair level of the expression profiles of TSSs even within a promoter. In contrast, the most commonly used high-throughput methodology for measuring gene expression, namely the microarray, profiles transcript expression without distinguishing between alternate 5' ends. Expressed sequence tag (EST) and full-length cDNA sequencing characterize end structures of transcripts, but their quantification ability is limited because of their cost. Additionally, some cDNA libraries are subtracted or normalized for exploration of novel transcripts, and these libraries cannot provide a quantitative view of expression [8,9].

In the FANTOM3 (functional annotation of mouse 3) project, the CAGE method was applied to more than 20 tissues from mouse and human [4,10]. More than seven million mouse CAGE tags were sequenced and mapped to the mouse genome, and so many core promoters are represented by many CAGE tags. This gives unprecedented opportunities to resolve the internal structures of core promoters.

As with cDNA sequencing, sequencing a large number of CAGE tags may capture errors, such as degraded transcripts or incomplete cDNA synthesis events. Extensive experimental and statistical validation of the CAGE set analyzed in this study, presented elsewhere (see the report by Carninci and coworkers [4] and its supplementary material), demonstrated good reliability even for single CAGE tags. A potential weakness with the method is the tag length (20-21 base pairs [bp]); with only a few sequencing errors, mapping tags back to the genome can be problematic. In the present study we used only unequivocal tag mappings [4] and focused on core promoters with more than 100 co-occurring tags. Another general issue with all tag-based technology is how to reliably associate tags with their corresponding full-length transcript; however, this is not a CAGE-specific problem and similar challenges are faced when using array-based methods.

Interestingly, transcription initiation was found to occur at multiple nucleotide positions within a core promoter region in many cases, although the start sites are more tightly clustered (but still not uniquely defined) for a subset of promoters with an over-representation of TATA boxes. Thereby, most core promoters do not have a single TSS but rather an array of closely located initiation sites. For clarity, this is conceptually different from alternative promoters, in which core promoters are separated by clear genomic space. In order to analyze arrays of tags corresponding to core promoters it is necessary to cluster adjacent tags [10]. A tag cluster is defined as a segment of a chromosome, on either the forward or reverse strand, where each 20 bp subregion contains at least one transcript 5' end identified by RIKEN full-length cDNAs, RIKEN-5' ESTs [10], GIS ditags [11], GSC ditags [12], or CAGE tags [7].

**Figure 1:**
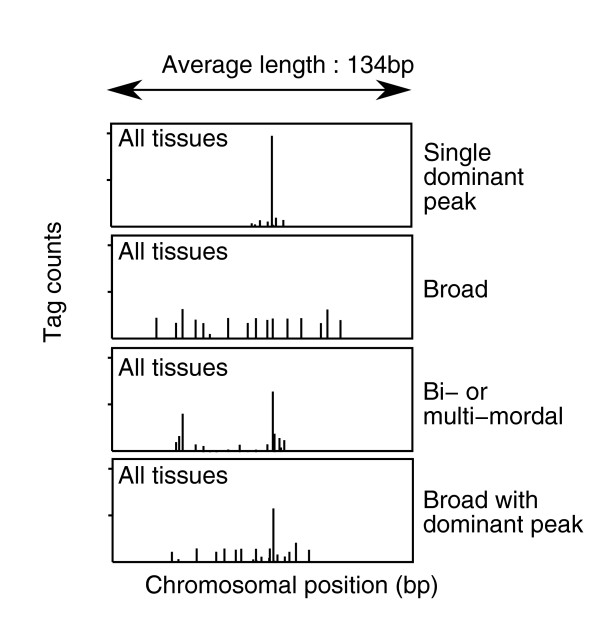
Four shape classes of static TSS usage. Tag clusters were classified into four classes based on CAGE tag counts from all tissues. The tag counts are displayed by histograms, where the x-axis indicates genomic coordinates or chromosomal location, and the y-axis indicates the total counts of CAGE tags. bp, base pairs; CAGE, cap analysis of gene expression; TSS, transcription start site.

We previously found that the TSS distributions of tag clusters have various 'shapes'. This means that there are various modes in selection of transcription initiation sites depending on promoters. In our previous study, tag clusters with sufficient (100 or more) CAGE tags for statistical analysis (1.1% [8,157] of the 736,403 tag clusters) were classified into four shape classes (for representative examples, see Figure 1): a single dominant peak (1,875 tag clusters), a general broad distribution (2,702), a broad distribution with a dominant peak (1,880), and a bimodal or multimodal distribution (1,700). Only the first class (23% of the 8,157) represents a narrowly defined TSS location, whereas the remaining classes are categories of broad regions with multiple TSSs. The single dominant peak class is associated with TATA boxes and tissue-specific expression, and the broad classes are associated with CpG islands and ubiquitous expression [4,10]. Although a classical model of transcriptional regulation can account for the single dominant peak class, it cannot explain arrays of TSS and their lack of TATA boxes. Because the shapes generally are very similar between human and mouse orthologous promoter regions, these properties strongly suggest that different modes of TSS selection exist between different promoter types [4].

A basic issue that must be addressed if we are to understand such broad transcription start regions is whether start site selection is precisely regulated or whether TSS usage is driven by nonspecific binding of basal transcription factors [13]. If TSS selection is regulated, then broad start regions could be caused by varying concentrations of transcription factors that favor initiation at different sites [14] or by epigenetic mechanisms such as DNA methylation, histone modifications, and chromatin remodeling [15-20]. If this is true, then it would be possible for the cell to modify the start site selection within a promoter in different contexts (such as tissues). On the other hand, if start site selection is primarily driven by the properties of the genomic sequence, then we would not expect major differences in TSS selection between tissues in a given broad promoter.

To address this issue, we examine tissue specificity at the base pair level, or fine-grained tissue-specific usage of TSSs. Note that our focus is not on alternative promoters, which are multiple promoters used by the same gene [4,21]. Rather, we investigate alternative TSSs within a core promoter region.

Here, we show that there are distinct, tissue-specific modes of start site selection within core promoters. To suggest possible mechanisms for this phenomenon, we show that such fine-grained tissue specificities of TSSs are associated with some expression contexts, such as tag cluster shapes, and genomic imprinting candidates.

## Results and discussion

### Tested tag clusters

We will be able to identify reliably only large usage biases if a tag cluster has few tags from each tissue, whereas more subtle biases will be reliably detectable if a tag cluster has many tags from some tissues. From this viewpoint, we use 8,157 tag clusters with 100 or more CAGE tags for statistical analysis. These clusters have previously been classified into the four shape classes based on CAGE tag distributions [4]. The mean length of these tag clusters is 134.2 bp, and 95% of them are under 250 bp in length. The mean lengths of the four classes based on their shapes or CAGE tag distributions are as follows: 87.0 bp for the single dominant peak, 146.7 bp for the broad distribution with a dominant peak, 180.5 bp for the multimodal distribution, and 129.1 bp for the general broad distribution. The mean length for the multimodal class is the longest among the four classes, being over twice the mean length for the single dominant peak.

**Figure 2:**
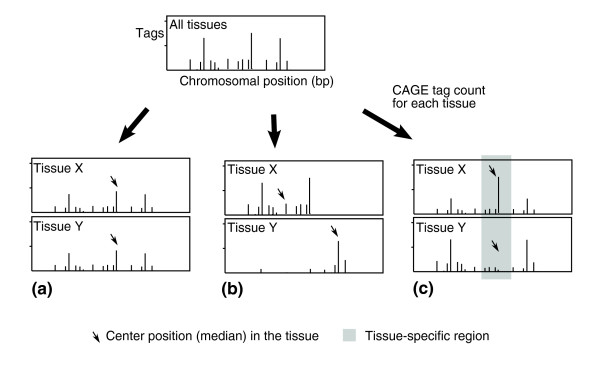
Possible cases of TSS usage among tissues. Possible cases of TSS usage sharing the same static structure of TSS: **(a) **TSS usage is identical between tissues X and Y; **(b) **upstream sites are favored in tissue X whereas downstream sites are favored in tissue Y; and **(c) **some subregions exhibit distinct TSS usage between tissues. The CAGE tag count of each tissue at each position is displayed as a vertical line, where the x-axis indicates genomic coordinates or chromosomal location and the y-axis indicates the CAGE tag count. bp, base pairs; CAGE, cap analysis of gene expression; TSS, transcription start site.

CAGE tags in a tag cluster come from several tissues, and their accumulation by each tissue and each genomic position is required to uncover dynamic usages of TSSs within a promoter. Figure 2 shows some possible cases of TSS selection within a promoter by different tissues, where panel a is a case of no differences between tissues, and panels b and c show cases of clear tissue specificity. Below, we examine whether the tag clusters have any tissue specificities, based on CAGE tag counts.

### Positionally biased promoters

In our exploration of tissue specificities within a tag cluster in which transcripts are initiated over a continuous region, we have no clear border to distinguish subregions to be compared with each other. The situation is different from exploration of alternative promoters, where each promoter is clearly separated by a certain genomic space. To cope with this issue, we adopt two strategies to explore fine-grained tissue specificity as comprehensively as possible: first, we explore differences in central (or median) TSS position depending on tissue; and second, we explore subregions whose expression profiles are different from the rest of the tag cluster. The first strategy can identify an intuitive type of fine-grained tissue specificity, namely overall bias of centered position, such as shown Figure 2b. There remain other types of tissue specificity, such as shows in Figure 2c, which has some internal regions with distinct tissue specificities but no clear differences in terms of the centered position. The second strategy was devised to find these cases.

**Figure 3:**
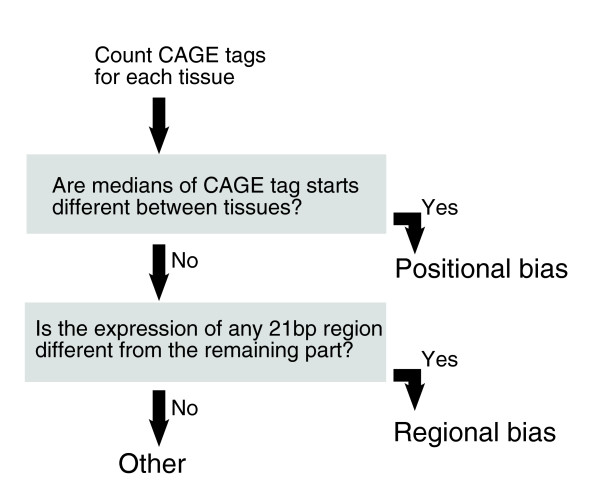
Classification of dynamic TSS usage within promoters. The classification flow of tag clusters. All of the examined tag clusters are classified into three categories - positional bias, regional bias, and others - based on CAGE tag counts from each tissue. bp, base pairs; CAGE, cap analysis of gene expression; TSS, transcription start site.

First, we examined whether the median location of transcription initiation within each tag cluster varies between tissues (Figure 3). This entails subdividing the tag cluster into multiple tag distributions depending on tissue, and then assessing whether the centers of all such tag distributions are similarly positioned. Because of the tag cluster definition, we would expect that some, if not all, of such subdistributions will overlap to some extent with each other, because if a group of tags does not overlap with any other then it would not be part of the initial cluster but would form a distinct alternative promoter. We did not attempt to fit the subdistributions to any generic template such as normal distributions, because the shapes can vary greatly [4] and in some cases there were too few tags to fit the subdistributions. Moreover, at the base pair level start site selection is biased toward pyrimidine-purine dinucleotides (where the transcript starts at the pyrimidine) [4], which makes any normality assumption unsound.

Given the above, we employed a statistical test with no in-built assumption about distributions, namely the Kruskal-Wallis one-way analysis of variance by ranks. It tests the null hypothesis that several samples come from populations with the same median [22] (this is essentially a nonparametric variant of the classical analysis of variance test). Thus, rejection of the null hypothesis implies that at least one of the underlying tag distributions has a distinct center point. The null hypothesis was rejected (*P *< 0.01) for 2,491 out of 8,157 tag clusters (30%), and we term these cases 'positionally biased'. The test does not indicate which tissues differ in median, just that they are not all the same.

**Figure 4:**
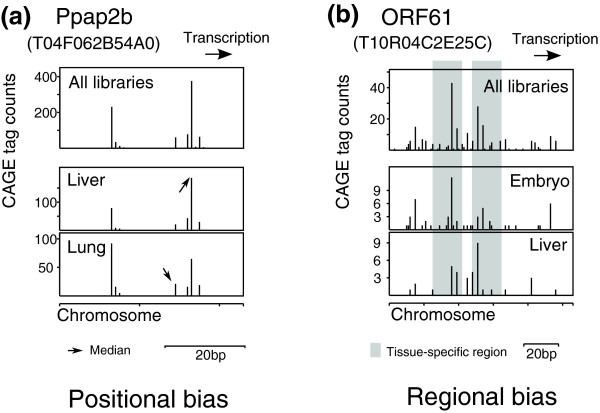
Examples of fine-grained tissue specificity. Examples of fine-grained tissue specificity are shown. **(a) **CAGE tag distribution of a tag cluster located at the 5' end of *Ppap2b *(tag cluster ID: T04F062B54A0), which is classified as positionally biased. The CAGE tag count at each nucleotide position is displayed as a vertical line. Totals of CAGE tag counts derived from all libraries are shown as all libraries, and two tissues picked from the 22 utilized tissues, liver, and lung are also shown. Arrows within the histograms indicate median locations of transcription initiation. **(b) **CAGE tag distribution of a tag cluster located at the 5' end of *ORF61 *(tag cluster ID: T10R04C2E25C), which is classified as regionally biased. The CAGE tag counts of all libraries, embryo, and liver are shown. Gray backgrounds indicate regions with different tissue specificity from the remaining part of the tag cluster. CAGE tag counts in each library and additional information for tag clusters is also accessible from the CAGE Analysis Database [39]. CAGE, cap analysis of gene expression.

An example of a positionally biased tag cluster is shown in Figure 4a. A tag cluster located at the 5' end of *PPap2b *(phosphatidic acid phosphatase type 2B) has two peaks of CAGE tags about 20 bp apart. The downstream peak is the most used and corresponds to the median in liver libraries, whereas the upstream peak is the most utilized in lung. These two regions are clearly utilized in a tissue-specific manner, and this results in a statistically significant difference in median TSS location. If TSS selection is influenced by distinct but proximal *cis *elements depending on tissues, then this type of TSS usage would be expected.

### Regionally biased promoters

Second, we identified tissue-specific subregions of 21 bp within tag clusters, using a Bayesian statistics based method developed previously for analysis of alternative splicing (see Materials and methods, below) [23].

**Figure 5:**
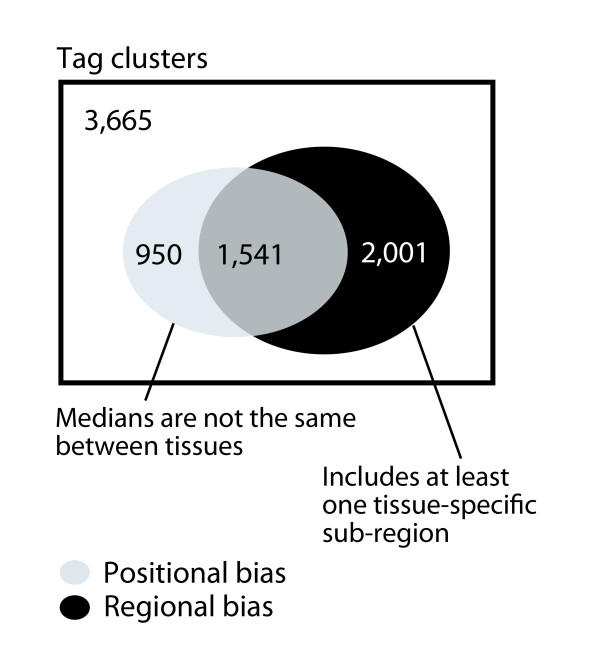
Tag clusters classified as positional and regional biases. The Venn diagram shows the number of tag clusters where start site selection is biased due to tissue. A biased tag cluster can either have distinct medians for tag starts for different tissues (termed positional bias) and/or have subregions that have a significantly different tag composition than the whole tag cluster. Most tag clusters that have positional bias also have subregions with significantly different tag composition. Only the tag clusters that are not classified as positional bias but include tissue-specific subregions are termed regional biases.

Of the total 8,157 tag clusters, 3,542 (43%) had at least one tissue-specific subregion. As expected, most of the positionally biased clusters (1,541/2,491 [62%]) also had tissue-specific subregions (Figure 5). In total, about half (4,492/8,157 [55%]) of the tag clusters examined exhibit internal tissue-specificity of some kind. Because the positionally biased clusters were already shown to have a tissue bias in TSS selection, we focused on those tag clusters that were not positionally biased but still had subregions with distinct tissue usage. We term these cases, which cover 2,001 out of 8,157 tag clusters (25%), 'regionally biased' (Figure 3).

An example of a regionally biased cluster is shown in Figure 4b. A tag cluster located at the 5' end of *ORF61*, which encodes a 574 amino acid protein of unknown function, has a broad shape, and the median TSS locations are positioned roughly in the center of the tag cluster. Although there is no significant difference of medians among tissues, the CAGE tag distributions in its subregions are different from each other depending on tissues. For example, upstream TSSs are used frequently in embryo whereas downstream TSSs are used frequently in liver. Tissue specificities change along the genome, but the other TSSs in the intermediate region and at both ends contribute to no significant difference in central TSS position.

### Associations with CpG islands and CAGE tag shape classes

To explore the context of promoters with dynamic TSS usage, we examined their relations with CpG islands. Of the 5,607 tag clusters located in CpG islands, 1,908 (34%) and 1,650 (29%) are classified as positionally and regionally biased, respectively. Table 1 shows associations between CpG islands, and positionally and regionally biased promoters. Each cell indicates a one-sided *P *value of the Fisher's exact test for the null hypothesis that the two categories do not have any positive association. For example, the cell in the first row and the first column indicates the result of the statistical test based on a 2 × 2 contingency table, whose columns represent positionally biased and other (not positionally biased) promoters and whose rows represent CpG and other (non-CpG) promoters. Table 1 indicates that both positionally and regionally biased tag clusters are associated with CpG islands with statistical significance (*P *< 1.0 × 10^-3^). Tag clusters containing internal regions with different tissue-specificities tend not to be in the single dominant peak class in which transcription starts from a narrowly fixed position. This is to some degree expected just because of the nature of the single dominant peak class, because the width of such promoters is small. These associations are consistent with the previous finding that broad tag clusters are associated with CpG islands [4].

We also examined their relations with shapes of CAGE tag distributions (Table 1). A significant association of positional bias with the multimodal shape class suggests that the multiple peaks are superimposed prominent TSSs utilized in a tissue-specific manner, implying that tag clusters with multimodal shapes consist of multiple and overlapping promoters. This can be expected from the definition of tag clusters, where two proximal and distinct promoters are joined if rarely used TSSs are located between them. Interestingly, Table 1 also shows a significant association of the regionally biased class with the general broad tag distribution. This reveals distinct tendencies between positional and regional biases, and that tag clusters without remarkable peaks are also regulated tissue specifically on a fine-grained scale. Nonspecific DNA binding of transcription factors [13] is unlikely to explain these tag clusters.

### Associations with imprinting

Genomic imprinting is epigenetic modification of genes whose expression is determined according to their parent of origin [24]. The key molecular mechanism is DNA methylation, which can repress transcription by direct and indirect mechanisms, such as inhibiting the binding of specific transcription factors, and recruiting methyl-CpG-binding proteins associated with repressive chromatin remodeling [25]. Interestingly, different machineries for maternal and paternal silencing have been suggested: maternal repression is effected by promoter methylation of a target transcript, and paternal repression by inactivation of its antisense transcript by maternal methylation [26]. Analysis of *Eed *mutant mice suggests that paternally and maternally inherited chromosomes can use different chromatin silencing mechanisms [27,28]; however, the details remain unclear.

To explore links between dynamic TSS usage and imprinting, we used candidate imprinted transcripts stored in the EICO database [29], which were identified by differential expression dependent upon chromosomal parent of origin using cDNA microarrays [30]. The sensitivity of the method was demonstrated by identification of previously reported imprinted genes [30]. It should be emphasized that the EICO database lists candidate imprinted transcripts and non-imprinted transcripts under the control of imprinted transcripts by identification of differential expression between parthenogenotes and androgenotes [30,31].

We found that 328 of the 8,157 tag clusters used in this study are located at 5' ends of the imprinting candidates, and 115 (35%) and 104 (31%) of them are classified as positionally and regionally biased, respectively. Table 1 shows the statistical significances of their associations with these candidates, which indicates that paternally and maternally imprinted transcripts are associated with positional and regional biases. We also found that paternal and maternal imprinting candidates are associated with the general broad shape class with *P *values of 0.04 and 1.6 × 10^-5^, where Fisher's exact test is used for the null hypothesis that paternal imprinting (or maternal imprinting) and the general broad shape class do not have any positive association. It is surprising that paternally imprinted promoters with positional bias are not associated with the multimodal shape class, which is a characteristic of positional bias in general. Although these paternally imprinted promoters are just special cases of positional bias, maternally imprinted promoters may be more representative cases of regional bias.

**Figure 6:**
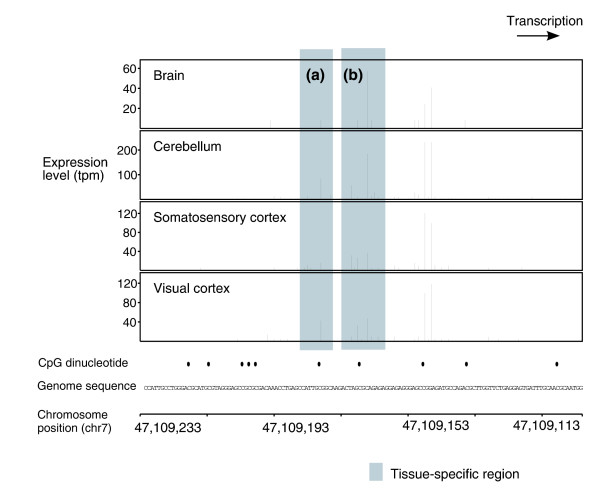
TSS usage and CpG dinucleotides at the 5' end of *Snrpn*. Expression levels of tag cluster T07R02CED41C, located at the 5' end of *Snrpn *on the reverse strand of chromosome 7, are shown. Only tissues with tpm (transcripts per million) above 100 and more than ten CAGE tags in the tag cluster are shown, and their expression levels are displayed as histograms. Gray backgrounds indicate regions with different tissue specificity from the remaining part of the tag cluster. Genome sequences are displayed below the graphs, and CpG sites are indicated by dots. CAGE, cap analysis of gene expression; TSS, transcription start site.

As an example, *Snrpn*, which encodes small nuclear ribonucleoprotein N, is an imprinted gene related to Prader-Willi syndrome, and its 5' end is maternally methylated [32]. The tag cluster T07R02CED41C is located at the 5' end of *Snrpn *and classified as regionally biased. Figure 6 shows the expression profile at the base pair level. Different tissue specificities can be observed in the regions with grey background. As seen in Figure 6, the B region, which exhibits high expression in general, is less expressed in somatosensory cortex and visual cortex, whereas the A region is less expressed in whole brain and somatosensory cortex. Methylation-sensitive polymerase chain reaction (PCR) has revealed that each of the CpG dinucleotides at the 5' end is methylated at different levels in the embryo, especially at 10.5 days *post coitum*, and also revealed that methylation levels change dynamically in a developmental process [33]. An interpretation of this fine-grained tissue specificity is that the differential methylation of each CpG dinucleotide affects the transcription machinery, and results in different specificities without a clear positional bias. This interpretation is based on the fact that specific paternal and maternal methylation of imprinted genes starts at different genomic locations and that CpG methylation gradients may influence transcription. This would affect fine-grained transcription start usage in a 'regionally biased' way among tissues for maternally imprinted genes.

### Associations with tissue-specific differentially methylated regions

Methylation is involved in tissue-specific expression in some cases, as well as genomic imprinting. Genome-wide analysis of DNA methylation status using restriction landmark genomic scanning (RLGS) [34] identified chromosomal regions that are differentially methylated in a tissue-specific manner [35,36]. Quantitative real-time PCR and bisulfite genomic sequencing revealed associations of DNA methylation with tissue-specific expression and partial methylation in some examples [36].

To explore the possibility that the fine-grained tissue specificities are associated with differential methylation, we compared these 150 differentially methylated regions identified by Song and coworkers [36] with our classification. Most of the regions are located at promoters and CpG islands, and 29 of the tag clusters used here overlap the differentially methylated regions. Of the 29 tag clusters, 13 (44%) and 11 (37%) are classified as positionally and regionally biased, respectively. These fractions are substantially larger than the fractions of all tag clusters (30% for positional bias and 25% for regional bias) and the fractions of CpG related tag clusters (34% for positional bias and 29% for regional bias), but additional data are required to prove the association with differential methylation rigorously. Given these initial results, we hypothesize that differences in DNA methylation due to cellular context is one of several mechanisms responsible for the observed difference in TSS selection between tissues.

## Conclusion

We found that TSSs are tissue-specifically utilized within a tag cluster, rather than uniformly among tissues, in about half of all tag clusters in this study. Tag clusters with multiple and prominent CAGE tag peaks and positionally biased tissue specificity can be interpreted as distinct and overlapping promoters. On the other hand, a substantial number of tag clusters contain broad TSSs with regionally biased tissue specificity. Although detailed understanding of their regulation will require further experimentation, our comparisons with genome imprinting candidates raise the hypothesis that some of these tissue-specific TSS usages are regulated via DNA methylation and/or subsequent chromatin remodeling.

Our study is based on a limited number of 22 tissues profiled by CAGE, and the number of tag clusters with fine-grained tissue specificities is bound to increase when more tissues and conditions are added. Our results give rise also to questions about TSSs and transcript 5' ends. In general, the transcripts with the most upstream 5' ends have been utilized to define TSSs of genes [37,38]. However, our findings imply that this methodology is biologically relevant only in some cases, because specific transcription starts frequently from nearby but distinct sites depending on tissue preferences. Comprehensive detection of TSSs in all tissues and conditions will be required to gain a complete understanding of transcriptional regulation and of the logic behind specific recruitment of transcription factors within core promoter elements.

This study highlights a property of core promoters that is little explored and less understood; it is clear that start site selection within promoters is a highly regulated process and that core promoters cannot be considered simply as standard templates serving to integrate signals from other *cis *regulatory elements.

## Materials and methods

### Data source

Mouse tag clusters and CAGE tag counts based on NCBI build 33 were retrieved from the CAGE Analysis Database [39], which provides CAGE tag counts for each library at the base pair level, associations of tag clusters with gene names, and additional information [40]. CAGE data belonging to different libraries from the same tissue were merged. Twenty-two tissues were used for our analysis (Table 2). Although some of them were not mutually exclusive, for example brain and cerebellum, they were treated as different categories. CpG island locations used in the above analysis are retrieved from the UCSC Genome Browser Database [41].

### Regionally biased tissue specificity

Here, we aimed to test the null hypothesis that a tag cluster does not contain any internal regions with different tissue specificity from the remaining part. Although a large number of CAGE tags are used, some regions inside tag clusters have few tags, because of our tag cluster definition stating that any region with at least one tag is a part of a tag cluster. To achieve a reliable test in cases with such a small number of CAGE tags, we used the tissue specificity score (TS) and the negative log value of its relative change (rTS), which was devised for finding tissue-specificity of alternative splicing from EST libraries [23]. Bayesian statistics is used to make a reliable detection even among tissues with small numbers of ESTs.

Call an internal region in a tag cluster R_int_, the remaining part R_rem_, a tissue T, and all of the other tissues U. Let the hidden or true frequency of CAGE tag counts in R_int _derived from T be θ_int,T_, and similarly for the other variables to yield θ_int,U_, θ_rem,T _and θ_rem,U_. They are normalized and should fulfill the following equations:

θ_int,T _+ θ_rem,T _= 1

θ_int,U _+ θ_rem,U _= 1

The tissue specificity score (TS) is calculated from the observed CAGE tag counts as follows:

TS = 100 (P [θ_int,T _> 0.5 | obs] - P [θ_rem,T _> 0.5 | obs])

Let the observed CAGE tag counts be N_int,T _in the internal region derived from T, and the negative log value of its relative change (rTS) is defined as follows:

rTS = -log10 (ΔTS/TS)

Where ΔTS = | TS(N_int,T_) - TS(N_int,T _- 1) |. A high TS score indicates that the internal region is much preferred in the tissue in comparison with the all of the other tissues, and a high rTS value indicates that the TS value is stable even if a single CAGE tag is not sequenced by chance.

This examination of the null hypothesis, that the internal region in the tag cluster does not exhibit different tissue specificity from the remaining part, is applied for each tissue. Each 21 bp subregion around a genome position where any CAGE tag alignment starts is tested, and the tested subregions can overlap. Because of this evaluation being conducted repeatedly in a tag cluster, we adopted more rigorous thresholds than were used in the original publication of this method, namely TS score above 90 and rTS score above 0.9.

### Statistical test for associations

Associations of the fine-grained tissue specificities with CpG islands, shapes of CAGE tag distribution, and genome imprinting candidates were evaluated by one-sided Fisher's exact test. A 2 × 2 contingency table for two sets of tag clusters is constructed, and the *P *value for the null hypothesis that the two sets do not have any positive association is evaluated.

## Additional data files

The following additional data are available with the online version of this paper. Additional file 1 is a document including all of our classifications of the tag clusters and their attributes in tab-delimited format.

## Supplementary Material

Additional file 1 The document includes all of our classifications of the tag clusters and their attributes in tab-delimited format. The first line indicates column names. The nonobvious columns are as follows: symbol, gene symbol associated with the tag cluster, where the associations were extracted from the CAGE Analysis Database [40]; IMPm and IMPf, candidate maternally and paternally imprinted transcripts; TDMbyRLGS, tissue-specific differentially methylated regions identified by Song and coworkers [36]. Click here for file

## Figures and Tables

**Table 1:** Statistical significance of associations with CpG islands, CAGE tag shape classes, promoter expression, and imprinting candidates

		Positional bias	Regional bias	Other
CpG islands	CpG	4.11 × 10^-25^*	1.65 × 10^-56^*	1.00
	Not CpG	1.00	1.00	2.22 × 10^-113^*
CAGE tag shape class	Single dominant peak	1.00	1.00	2.13 × 10^-182^*
	Broad distribution with a dominant peak	1.92 × 10^-02^	3.72 × 10^-01^	9.89 × 10^-01^
	Multimodal Distribution	2.37 × 10^-12^*	1.49 × 10^-02^	1.00
	General broad Distribution	6.70 × 10^-01^	3.19 × 10^-79^*	1.00
Imprinting candidates	Maternal imprinting candidates	7.94 × 10^-01^	1.86 × 10^-04^*	9.97 × 10^-01^
	Paternal imprinting candidates	6.65 × 10^-04^*	2.77 × 10^-01^	1.00
	Not candidates	9.69 × 10^-01^	9.99 × 10^-01^	7.08 × 10^-06^*

Shown are the levels of statistical significance (*P *values) of associations with CpG islands, CAGE tag shape classes, promoter expression, and imprinting candidates, where the Fisher's exact test is used for the null hypothesis that the two examined sets do not have any positive associations. *Statistically significant findings. CAGE, cap analysis of gene expression.

**Table 2:** Utilized tissues and their CAGE tag counts in mouse

Tissue	Total CAGE tags
Adipose	237,434
Amnion	381
Brain	122,603
Cerebellum	211,541
Cerebral cortex	17,510
Diencephalon	43,044
Embryo	1,077,740
Eye	1,246
Heart	34,743
Hippocampus	2,728
Liver	2,130,614
Lung	1,129,877
Macrophage	1,231,138
Mammary gland	1,294
Medulla oblongata	3,193
Muscle	42,294
Placenta	249
Prostate gland	57,057
Somatosensory cortex	190,762
Striatal primordia	39,693
Testis	109,150
Visual cortex	211,620

CAGE, cap analysis of gene expression.
